# Transmembrane Protein TMEM59L Modulates 5‐FU Resistance via PTPRN‐Mediated DNA Damage Repair in Colorectal Cancer

**DOI:** 10.1002/cnr2.70448

**Published:** 2026-01-16

**Authors:** Wenzhi Jin, Qiang Ma, Zenghui Ma, Jianhua Hou, Fenming Wang, Huiqing Kang, Chen Wang, Xiaoliang Wang, Feng Liu

**Affiliations:** ^1^ Department of Hepatobiliary Surgery Pudong Hospital Affiliated to Fudan University Shanghai China; ^2^ Department of General Surgery The People's Hospital of Wuhai Inner Mongolia Wuhai Inner Mongolia China; ^3^ Department of Pediatric Surgery Children's Hospital of Fudan University, National Children's Medical Center Shanghai China

**Keywords:** colorectal cancer, DNA damage repair, drug resistance mechanisms, TMEM59L, transmembrane protein

## Abstract

**Background:**

Chemotherapy resistance in colorectal cancer (CRC) is often mediated by enhanced DNA damage repair (DDR). Transmembrane protein TMEM59L is implicated in cancer progression, but its role in CRC chemoresistance is unclear. We investigated whether TMEM59L regulates 5‐fluorouracil (5‐FU) sensitivity through PTPRN‐mediated DDR.

**Aims:**

This study aimed to investigate the role of TMEM59L in regulating PTPRN‐mediated DNA damage repair and its impact on 5‐FU sensitivity in colorectal cancer, with the goal of identifying potential therapeutic targets to overcome chemoresistance.

**Methods and Results:**

Bioinformatics analyses assessed TMEM59L/PTPRN expression and prognosis in CRC cohorts. Gain‐ and loss‐of‐function experiments were conducted in CRC cell lines (HCT116, SW480) and their 5‐FU‐resistant derivatives. Cell proliferation, migration, invasion, apoptosis, DNA damage, and reactive oxygen species (ROS) were measured. Protein interactions were examined by Western blot and immunofluorescence. A xenograft model in nude mice validated the TMEM59L/PTPRN axis on tumor growth, stemness, and EMT markers. TMEM59L expression was elevated in metastatic lesions and associated with poor CRC patient survival. Functionally, TMEM59L promoted malignant behaviors and epithelial–mesenchymal transition. It was upregulated in 5‐FU‐resistant cells and non‐responsive patients. TMEM59L knockdown sensitized cells to 5‐FU, increasing ROS, DNA damage, and apoptosis, while its overexpression induced resistance. Mechanistically, TMEM59L regulated 5‐FU‐induced DNA damage and ROS through PTPRN. PTPRN overexpression reversed the sensitization caused by TMEM59L silencing. In vivo, TMEM59L knockdown enhanced 5‐FU's antitumor effect, which was counteracted by PTPRN overexpression.

**Conclusion:**

The TMEM59L/PTPRN axis is a key regulator of DDR and 5‐FU resistance in CRC. TMEM59L promotes chemoresistance via PTPRN by enhancing DNA repair and reducing ROS‐mediated apoptosis. Targeting this pathway may offer a novel strategy to overcome 5‐FU resistance.

**Abbreviations**
5‐FU: 5‐fluorouracil
CCK‐8: Cell Counting Kit‐8
COAD: colon adenocarcinoma
CRC: colorectal cancer
EMT: epithelial‐mesenchymal transition
GBM: glioblastoma
HRP: horseradish peroxidase
IHC: immunohistochemistry
NC: negative control
PI: propidium iodide
READ: rectal adenocarcinoma
ROS: reactive oxygen species
SP: streptavidin peroxidase
TMB: tumor mutation load
TMZ: temozolomide

## 1 Introduction

Colorectal cancer, a disease with high morbidity and mortality, continues to threaten global health. It ranks as the third most commonly diagnosed cancer in men and the second in women worldwide [1]. The factors influencing CRC incidence and patient outcomes are complex, with chemotherapy sensitivity—closely tied to DNA damage and repair mechanisms—playing a particularly important role [2, 3]. Indeed, chemotherapy response in CRC is frequently associated with DNA damage and repair capacity [3, 4].

TMEM59L is a transmembrane protein belonging to the membrane transport protein family, members of which are increasingly recognized as key regulators of chemoresistance in multiple cancers [5]. Although not classic solute carriers, TMEM family proteins are involved in vesicular trafficking, ion homeostasis, and signaling pathways related to drug uptake and efflux. TMEM59L expression has been significantly correlated with immunophenoscore, neoantigen load, loss of heterozygosity, cancer stemness, and homologous recombination deficiency across various malignancies [5]. In glioblastoma (GBM), TMEM59L is highly expressed in recurrent tumors but low in normal brain tissue. Radiation has been shown to induce TMEM59L expression in GBM cells, and interestingly, high TMEM59L levels are associated with better prognosis in IDH‐mutant and MGMT‐methylated gliomas, suggesting a possible connection with DNA damage repair and oxidative stress [6]. Oxidative stress can promote DNA strand breaks and genomic instability; notably, radiotherapy induces DNA damage largely through reactive oxygen species [6]. Thus, elevated TMEM59L expression may enhance radiosensitivity by amplifying ROS‐mediated DNA damage and impairing repair mechanisms. In CRC, TMEM59L has been identified as an independent prognostic factor in patients with lymph node metastasis, along with CLCA1 and TUBB2B [7].

Studies indicate that both PTPRN and RIM‐BP2 are downregulated in GBM tissues compared to normal brain tissue, and Kaplan–Meier analysis suggests their potential utility as prognostic biomarkers [8]. Notably, PTPRN expression is significantly elevated in patients resistant to radiotherapy, though not associated with temozolomide (TMZ) resistance [8]. Tumors with low PTPRN expression also exhibit higher tumor mutation burden (TMB) and increased immune cell infiltration compared to those with high PTPRN levels [8]. In low‐grade gliomas, PTPRN has been proposed as a prognostic marker linked to immune infiltration [9], whereas high PTPRN expression correlates with poor outcomes in high‐grade glioma [10]. In CRC, elevated PTPRN is associated with reduced survival, and its knockdown has been shown to suppress invasiveness in vitro and reduce liver metastasis in vivo by modulating epithelial–mesenchymal transition and impairing insulin receptor signaling [11].

5‐Fluorouracil (5‐FU) remains a cornerstone of CRC chemotherapy. Its efficacy depends on intracellular folate cofactors and DNA damage repair activity; resistant cells may evade 5‐FU–induced DNA damage by switching to mitochondrial serine metabolism to support purine synthesis [12].

In this study, we aimed to investigate the role of TMEM59L in regulating PTPRN‐mediated DNA damage repair and its impact on 5‐FU sensitivity in CRC. Using bioinformatics analysis, in vitro experiments, and in vivo models, we sought to clarify the underlying mechanisms and identify potential therapeutic targets to enhance chemosensitivity in colorectal cancer.

## 2 Materials and Methods

### 2.1 Bioinformatics Approaches

The expression levels of TMEM59L and PTPRN in colorectal cancer (CRC) patients were investigated using tumor and normal tissue data from TCGA‐COAD (275 tumor vs. 349 normal samples) and TCGA‐READ (92 tumor vs. 318 normal samples) databases through the GEPIA analytical platform. Survival outcomes were evaluated via the Kaplan–Meier Plotter tool. GeneMANIA network analysis was employed to predict potential target genes interacting with TMEM59L, supplemented by correlation analysis to identify associated genes. Therapeutic response data extracted from the ROC Plotter database revealed significant differential expression of TMEM59L between treatment non‐responders and responders across three chemotherapy regimens: 5‐FU (279 vs. 379 samples, *p* = 0.0002), oxaliplatin (173 vs. 265 samples, *p* = 0.012), and capecitabine (62 vs. 47 samples, *p* = 1.9 × 10^−5^). This multi‐database approach integrated genomic expression profiling with clinical outcome assessments to characterize TMEM59L's potential role in CRC progression and treatment resistance.

### 2.2 Clinic Samples and Cell Lines

Twelve matched tumor and adjacent normal tissue samples were obtained from colorectal cancer (CRC) patients at Pudong Hospital Affiliated to Fudan University with written informed consent from all participants. The study received approval from the hospital's Ethics Committee and adhered to the Declaration of Helsinki and applicable national regulations. Five human CRC cell lines (NCI‐H716, HCT116, COLO 320DM, SW48, SW480) were sourced from the American Type Culture Collection (ATCC, USA), while a 5‐fluorouracil‐resistant HCT116 subline (HCT116/FU, BNCC342640) was obtained from BNCC (China). To maintain their chemoresistant phenotype, these cells were cultured in RPMI‐1640 medium with 10% fetal bovine serum (FBS), 1% penicillin‐streptomycin, and 25 μg/mL 5‐FU. Additionally, a 5‐FU‐resistant SW480 cell line (SW480/5FU) acquired from Changsha Aibiwei Biological Technology was cultured in L15 medium containing 10% FBS, 1% penicillin‐streptomycin, and 25 μg/mL 5‐FU.

### 2.3 Western Blot

Proteins were extracted using RIPA lysis buffer on ice, and concentrations were measured with a BCA assay kit. After separation by SDS‐PAGE, proteins were transferred onto PVDF membranes, which were then blocked for 1 h at room temperature using 5% skim milk in TBST to prevent non‐specific binding. The membranes were incubated overnight at 4°C with primary antibodies against TMEM59L (1:1000, Novus), CD133 (1:1000, Zenbio), SOX2 (1:800, Zenbio), Oct4 (1:1000, Zenbio), ABCG2 (1:500, Zenbio), γ‐H2AX (1:1000, Zenbio), and GAPDH (1:2000, Zenbio). Following three washes with TBST, HRP‐conjugated secondary antibodies (goat anti‐rabbit or anti‐mouse IgG H&L, 1:5000, Zenbio) were applied for 1 h at room temperature. Protein signals were visualized using an enhanced chemiluminescence detection system.

### 2.4 Immunohistochemistry (IHC) Analysis

Formalin‐fixed paraffin‐embedded specimens from human CRC clinical samples and HCT116 xenograft tumors (4% paraformaldehyde, 72 h fixation) were sectioned at 4 μm thickness for immunohistochemical analysis. Tissue sections underwent antigen retrieval followed by sequential incubation with primary antibodies targeting TMEM59L (bs‐11648R, Bioss), Ki67 (28074‐1‐AP), CD133 (252208), E‐cadherin (20874‐1‐AP), and Vimentin (10366‐1‐AP) (Proteintech/ZENBIO as specified). A biotin‐streptavidin‐HRP detection system employing species‐matched secondary antibodies was applied at 37°C for 30 min, with chromogenic development achieved through DAB substrate exposure. Counterstaining with hematoxylin preceded progressive ethanol dehydration and xylene clearing, with final mounted sections analyzed under bright‐field microscopy.

### 2.5 Cell Transfection

TMEM59L, PTPRN overexpression vector and corresponding negative control (oe‐NC), TMEM59L, PTPRN silencing vector and corresponding negative control (sh‐NC) were purchased from Yuanke Biotechnology Co. Ltd (China). These vectors were transfected into HCT116, HCT116/FU, SW480, and SW480/FU, respectively, using the Lipofectamine 8000 kit.

### 2.6 Drug Sensitivity Assay

For cytotoxicity assessment, stable TMEM59L‐knockdown and overexpression models of HCT116/SW480 colorectal cancer cells were plated in triplicate at 3000 cells/well. Following 24‐h exposure to 5‐FU concentration gradients, cellular responses were quantified using CCK‐8 reagent (C0038, Beyotime) through 90‐min chromogenic conversion. Drug susceptibility profiling revealed distinct IC50 patterns between cell subtypes—both lines tested at 25 μg/mL 5‐FU concentrations. Viability metrics were calculated as percentage ratios relative to untreated counterparts.

### 2.7 EdU Staining

For cell proliferation analysis, cultures were established in 24‐well plates at 5 × 10^5^ cells/well with 24‐h stabilization. EdU incorporation (50 μM, C0078S kit, Beyotime) proceeded for 2 h prior to sequential processing: PBS washing, 4% paraformaldehyde fixation (30 min), and 0.5% Triton X‐100 membrane permeabilization (10 min). Fluorescent detection involved sequential 30‐min incubations—first with Apollo reaction mixture (200 μL) for EdU visualization, followed by Hoechst 33342 nuclear counterstain (200 μL), both protected from light at ambient temperature. Quantitative assessment involved fluorescence microscopy imaging (Nikon Eclipse Ti) with proliferation rates determined through systematic counting of EdU‐positive nuclei across five representative microscopic fields per replicate.

### 2.8 Apoptosis Assay Through Flow Cytometry

To assess apoptosis, transfected SW480 and HCT116 cells in the logarithmic growth phase were treated with serum‐free RPMI‐1640 medium containing 25 μg/mL 5‐FU for 48 h. After treatment, cells were washed with PBS, adjusted to a density of 1 × 10^6^ cells/mL, and stained using the Annexin V‐FITC/PI Apoptosis Detection Kit (Thermo Fisher Scientific). Each sample was incubated with 5 μL Annexin V‐FITC and 10 μL propidium iodide (PI) in 100 μL binding buffer for 15 min at room temperature, protected from light. Apoptotic profiles were analyzed by flow cytometry (BD FACSCalibur), with cell populations categorized as viable (Annexin V^−^/PI^−^), early apoptotic (Annexin V^+^/PI^−^), late apoptotic (Annexin V^+^/PI^+^), or necrotic (Annexin V^−^/PI^+^), based on fluorescence gating established through appropriate controls.

### 2.9 Colony Formation Assay

Clonogenic survival analysis was performed on TMEM59L‐silenced cell pairs. Cellular suspensions containing 200 viable cells were seeded into 6‐well culture plates and maintained under standard culture conditions (37°C, 5% CO_2_) for 14 days to permit colony development. Following methanol‐free fixation with 4% paraformaldehyde, cellular aggregates were stained with 0.05% crystal violet solution for quantitative analysis. The number of colonies (> 50 cells/colony) was counted under a light microscope.

### 2.10 Immunofluorescence

Immunofluorescence characterization was performed on acid‐etched coverslip‐adherent cultures of HCT116 and SW480 lines (5 × 10^4^ cells/slip). Following 24‐h adhesion stabilization, samples underwent sequential processing: PBS washing, 4% paraformaldehyde fixation (30 min, ambient temperature), and 0.1% Triton X‐100 permeabilization (15 min). Non‐specific binding sites were blocked with 3% BSA prior to overnight incubation at 4°C with primary antibodies targeting DNA damage marker p‐γ‐H2AX (ab81299, Abcam) and EMT regulators E‐cadherin (20874‐1‐AP)/Vimentin (10366‐1‐AP, Proteintech). Alexa Fluor‐conjugated secondary antibodies (Invitrogen, 1:500) were applied for 60 min at 25°C followed by nuclear counterstaining with DAPI (5 min). Multiplex fluorescence signals were captured using a confocal laser‐scanning microscope (Leica TCS SP8), with z‐stack acquisitions processed through deconvolution algorithms to minimize out‐of‐focus light interference.

### 2.11 Construction of the Tumor Xenograft Model

The experimental protocol employed 5‐week‐old male BALB/c nude mice (18–20 g, Weitong Lihua, Beijing). Following one‐week acclimatization, animals were randomized into treatment cohorts (*n* = 5/group) for orthotopic xenograft establishment. Chemoresistant HCT116/FU cells (1 × 10^6^) suspended in 100 μL PBS containing 50% Matrigengel matrix (Corning) were unilaterally injected into the fourth mammary fat pads. Tumor progression was monitored through weekly bi‐dimensional measurements using digital calipers, with volumetric calculations performed as *V* = 0.5 × (long axis) × (short axis)^2^. Throughout the experiment, the investigator remained unaware of the group assignments of the animals to ensure blinding. The sample sizes used in each experiment are detailed in the corresponding figure legends, and all collected data were included in the analysis without any exclusions.

### 2.12 Statistical Analysis

Data analysis was conducted using SPSS version 20.0 (SPSS Inc., Chicago, IL). Results are expressed as the mean ± standard deviation (SD) based on a minimum of three independent experiments. For comparisons involving three or more groups, one‐way analysis of variance (ANOVA) followed by the Bonferroni post hoc test was applied. A *p* value less than 0.05 was considered to indicate statistical significance.

## 3 Results

### 3.1 Upregulation of TMEM59L Predicts Poor Prognosis for Patients With Colorectal Cancer

**FIGURE 1:**
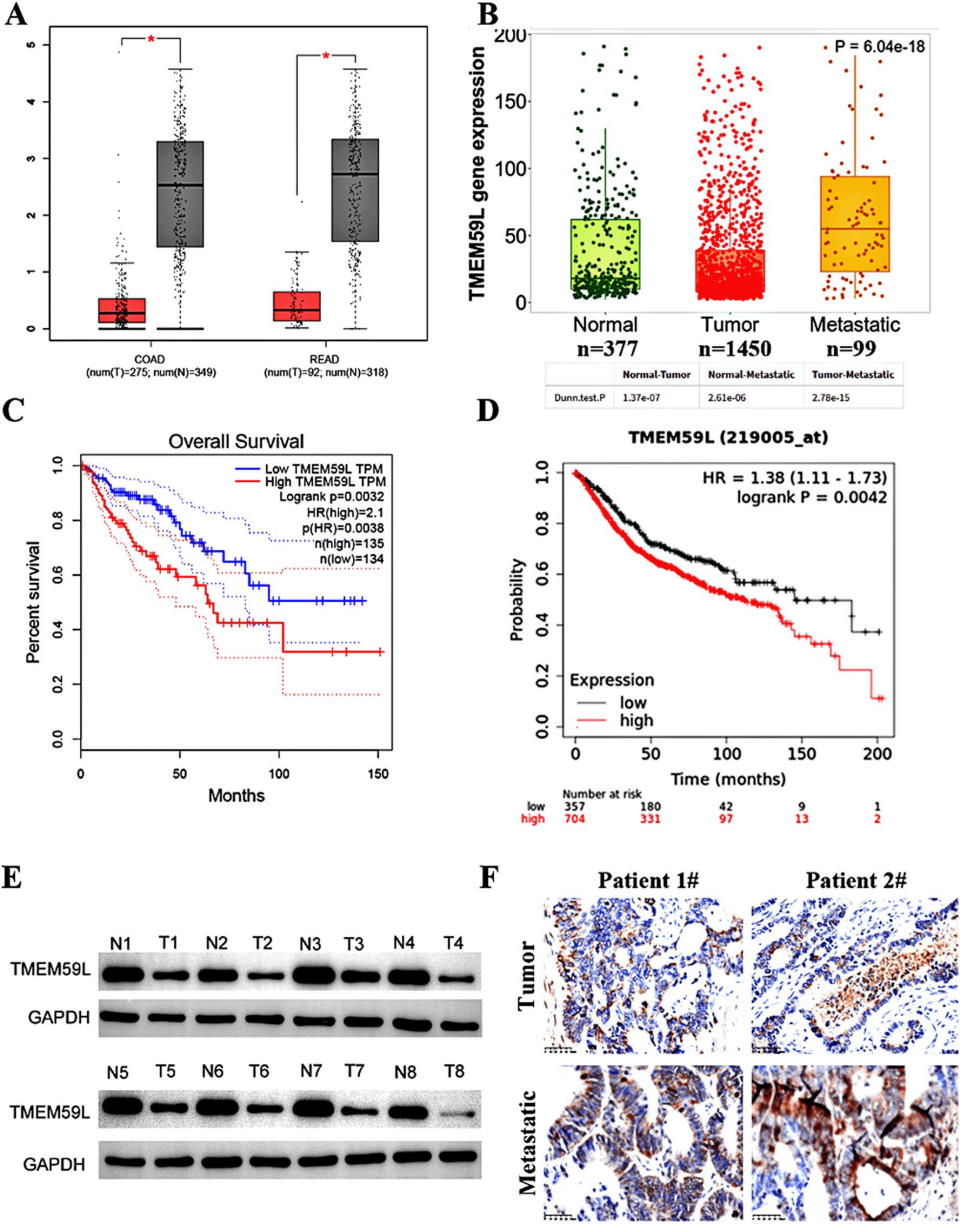
Upregulation of TMEM59L predicts poor prognosis for patients with colorectal cancer. (A) TMEM59L expression in COAD and READ from the TCGA database analyzed by the GEPIA database. (B) TMEM59L expression analysis in Normal, Tumor and Metastatic CRC tissues in the TNMplot database. (C, D) Higher TMEM59L expression was related to poorer OS in CRC patients from TCGA through GEPIA2 and Kaplan–Meier Plotter database. (E) Western blotting to determine TMEM59L expression in paired CRC and normal tissues, *n* = 8. (F) Immunofluorescence staining of TMEM59L in paired CRC and hepatic metastases tissues, *n* = 2.

Bioinformatics analysis revealed an unexpected pattern of TMEM59L expression: it was higher in normal colorectal tissues than in tumor tissues (Figure 1A,B). However, TMEM59L expression was markedly elevated in metastatic tumors compared to both primary tumors and normal tissues (Figure 1B). Interestingly, although the gene is downregulated in primary tumors, survival analysis showed that CRC patients with higher TMEM59L expression had significantly worse overall survival than those with lower expression (Figure 1C,D; *p* < 0.05). These findings were corroborated at the protein level: Western blotting confirmed lower TMEM59L expression in tumor tissues relative to normal tissues, while immunohistochemical staining revealed a pronounced increase in TMEM59L expression in distant metastases compared to primary tumor samples (Figure 1E,F).

### 3.2 TMEM59L Regulates Colorectal Cancer Cells Proliferation, Migration and Invasion

**FIGURE 2:**
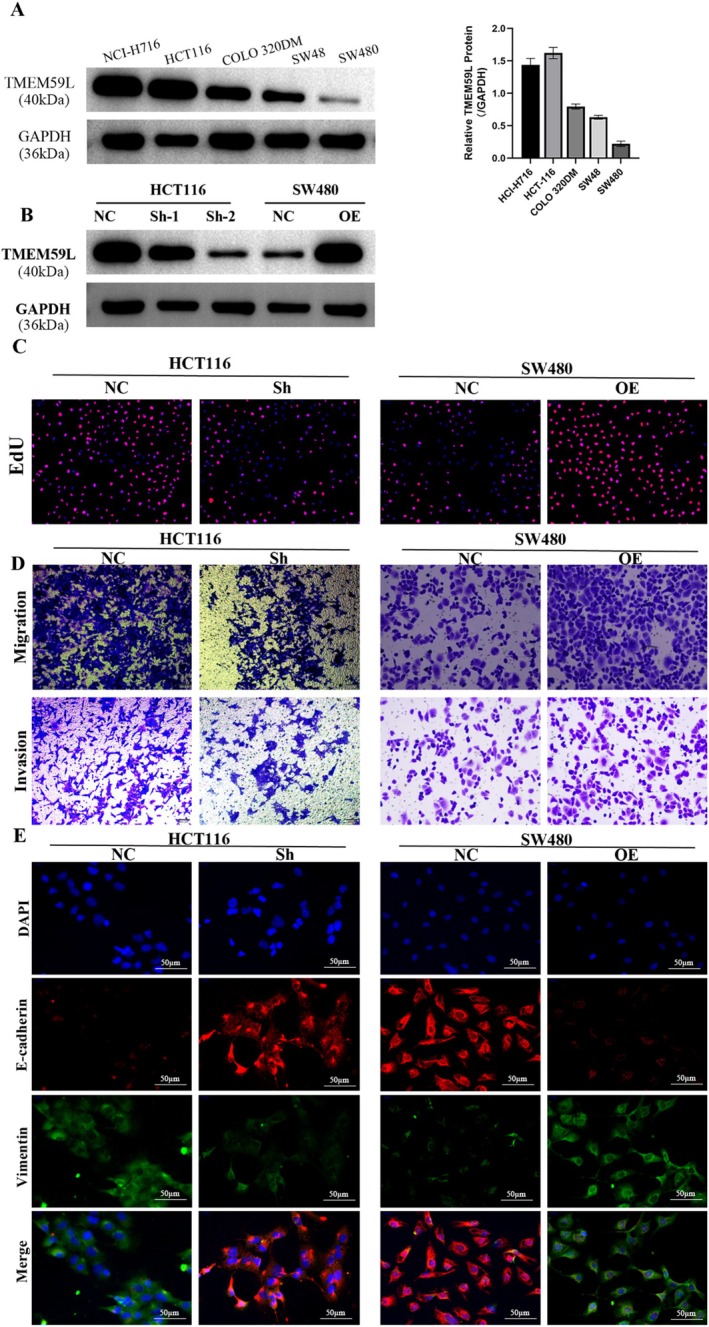
TMEM59L regulates colorectal cancer cells proliferation, migration, and invasion. (A) Western blotting confirmed expression of TMEM59L in different CRC cell lines. (B) Western blotting detects the knockdown of TMEM59L by shRNA and overexpression of TMEM59L by plasmid. (C) Downregulation of TMEM59L suppresses cell proliferation in HCT116 cells; overexpression of TMEM59L in SW480 promotes cell proliferation. (D) The function of TMEM59L on the migration and invasion ability of CRC cells was detected by Transwell assay. (E) E‐cadherin and Vimentin were evaluated through immunofluorescence staining in TMEM59L knockdown and overexpression CRC cells.

To investigate the role of TMEM59L in CRC cell proliferation, migration, and invasion, we first measured its baseline expression in several CRC cell lines. As shown in Figure 2A, TMEM59L expression was relatively high in HCT116 cells and low in SW480 cells compared to other lines. Based on this, HCT116 was selected for knockdown experiments and SW480 for overexpression studies; transfection efficiency was confirmed in Figure 2B. TMEM59L knockdown in HCT116 cells significantly suppressed proliferation, migration, and invasion (Figure 2C,D), whereas TMEM59L overexpression in SW480 cells enhanced these malignant behaviors. Consistent with these findings, epithelial–mesenchymal transition (EMT) marker analysis showed that E‐cadherin expression increased upon TMEM59L silencing in HCT116 cells but decreased upon TMEM59L overexpression in SW480 cells (Figure 2E). Vimentin expression exhibited the opposite trend, rising with TMEM59L overexpression and falling with its knockdown.

### 3.3 TMEM59L Was Elevated in 5‐FU Resistance CRC Cell Lines and Reduced 5‐FU Sensitivity

**FIGURE 3:**
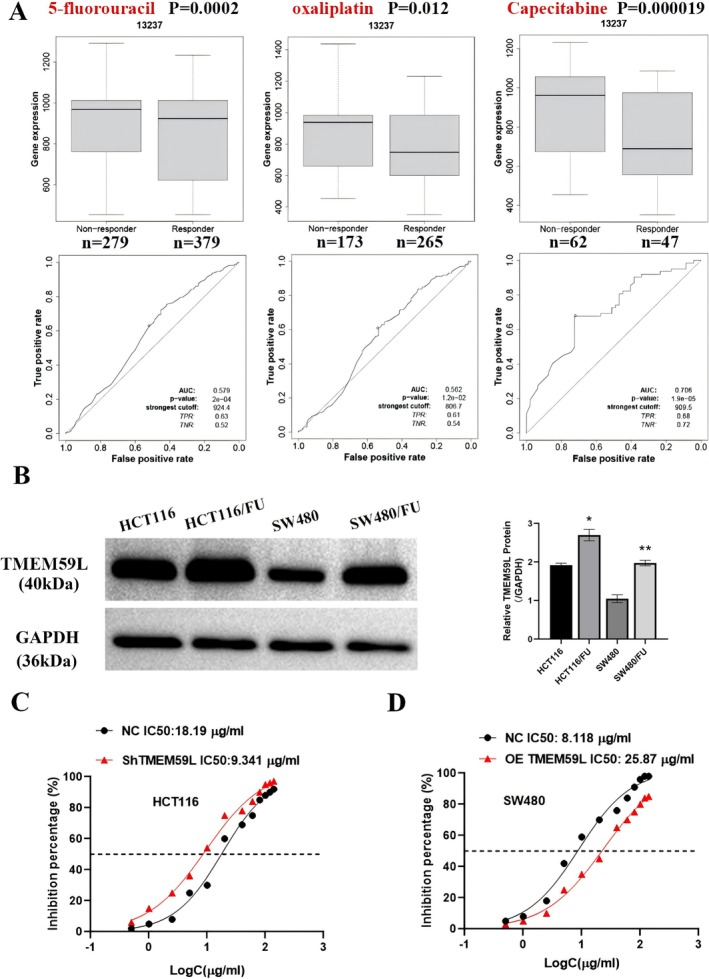
TMEM59L was elevated in 5‐FU resistance CRC cell lines and reduced 5‐FU sensitivity. (A) The expression of TMEM59L in non‐responder and responder groups treated by 5‐FU (*n* = 279 vs. 379, *p* = 0.0002), oxaliplatin (*n* = 173 vs. 265, *p* = 0.012) and Capecitabine (*n* = 62 vs. 47, *p* = 0.000019), respectively. (B) TMEM59L level in CRC cell lines and corresponding 5‐FU resistant cells was examined by western blot. (C, D) CCK‐8 assays of TMEM59L downregulation and upregulation on sensitivity of HCT116 and SW480 cells to 5‐FU; the half maximal inhibitory concentration (IC50) was calculated using GraphPad software. Data represent the mean ± SD (*n* = 3), *p < 0.05 vs. HCT116 group, ^$^ *p* < 0.05 vs. SW480 group.

We next assessed the relationship between TMEM59L and chemotherapy sensitivity. Bioinformatics analysis indicated that TMEM59L expression was elevated in CRC patients who did not respond to 5‐FU, oxaliplatin, or capecitabine, compared to those who did (Figure 3A). We then established 5‐FU‐resistant HCT116 and SW480 cell lines and observed upregulated TMEM59L expression in both (Figure 3B). Cell viability assays revealed that TMEM59L knockdown in HCT116 cells reduced the IC₅₀ of 5‐FU by half (from 18.19 to 9.341 μg/mL), while TMEM59L overexpression in SW480 cells increased the IC₅₀ twofold (from 8.118 to 25.87 μg/mL) (Figure 3C,D). These results suggest that TMEM59L significantly enhances 5‐FU resistance in CRC cells.

### 3.4 Silencing of TMEM59L Enhanced DNA Damage and 5‐FU Sensibility in Colorectal Cancer Cells and Drug‐Resistant CRC Cell Lines

**FIGURE 4:**
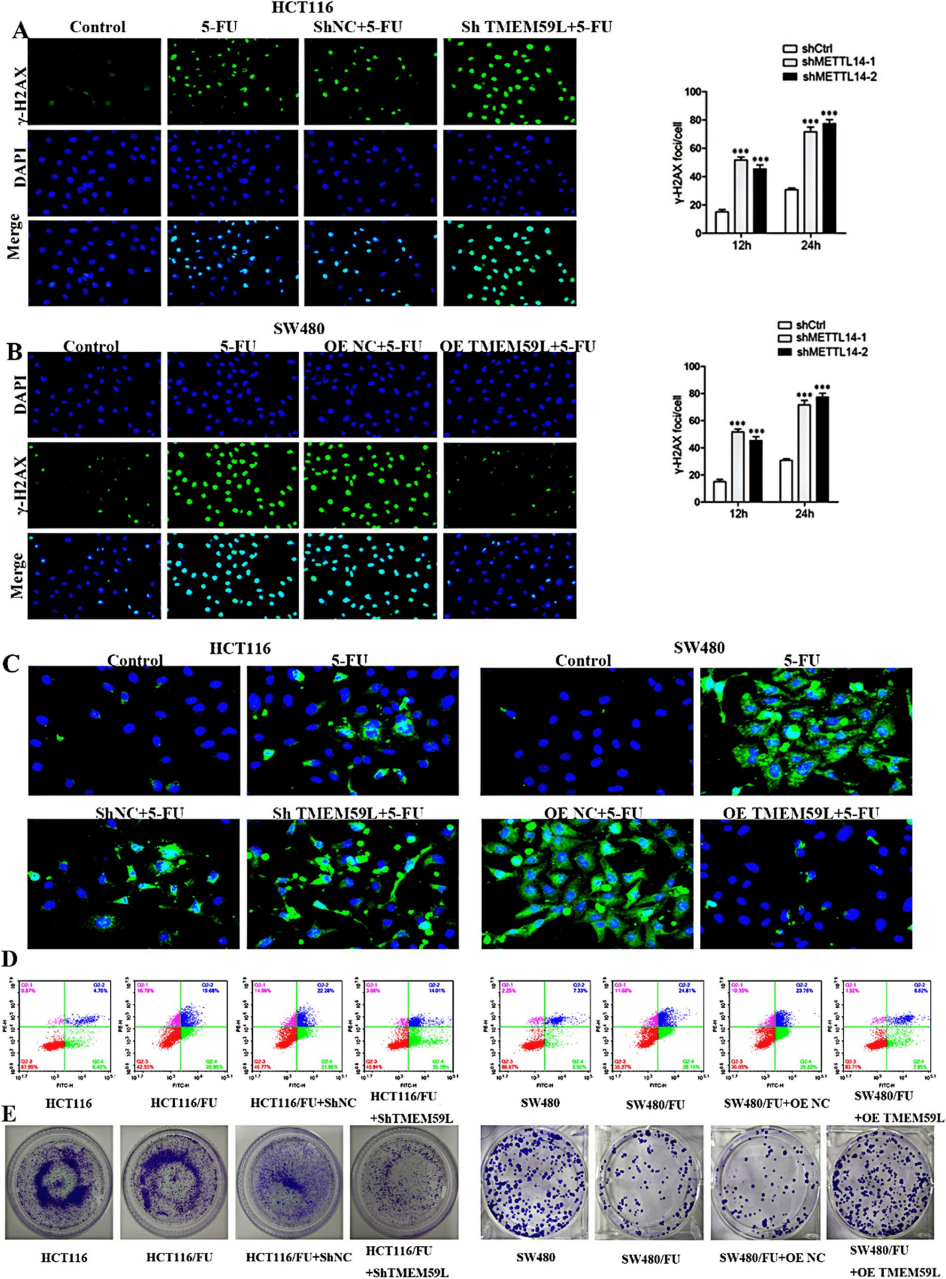
Silencing of TMEM59L enhanced DNA damage and 5‐FU sensibility in colorectal cancer cells and drug‐resistant CRC cell lines. (A, B) γ‐H2AX foci formation in HCT116 and SW480 cells was detected by immunofluorescence 48 h after treatment with 5‐FU (25 μg/mL). (C) Intracellular ROS levels in HCT116 and SW480 cells treated with 5‐FU for 48 h were detected by reactive oxygen species detection kit. (D) Effect of TMEM59L on apoptosis in CRC cells induced by 5‐FU (25 μg/mL) treatment for 48 h was determined by flow cytometric analysis. (E) Downregulation of TMEM59L reduced colony formation of HCT116/FU and SW480/FU cells.

We further evaluated the influence of TMEM59L on 5‐FU–induced DNA damage. As shown in Figure 4A,B, 5‐FU treatment increased γ‐H2AX expression, a DNA damage marker, in CRC cells. TMEM59L knockdown enhanced this effect in HCT116 cells, whereas TMEM59L overexpression attenuated it in SW480 cells. Given the link between ROS and DNA damage, we measured ROS levels and found that 5‐FU–induced ROS accumulation was promoted by TMEM59L knockdown but suppressed by its overexpression (Figure 4C). Apoptosis assays showed that low TMEM59L expression sensitized HCT116 cells to 5‐FU–induced apoptosis, while high TMEM59L expression protected SW480 cells from 5‐FU–induced cell death (Figure 4D). Colony formation assays confirmed that TMEM59L silencing significantly reduced 5‐FU resistance in CRC cells (Figure 4E).

### 3.5 TMEM59L Regulated 5‐FU Induced DNA Damage and ROS Through PTPRN

**FIGURE 5:**
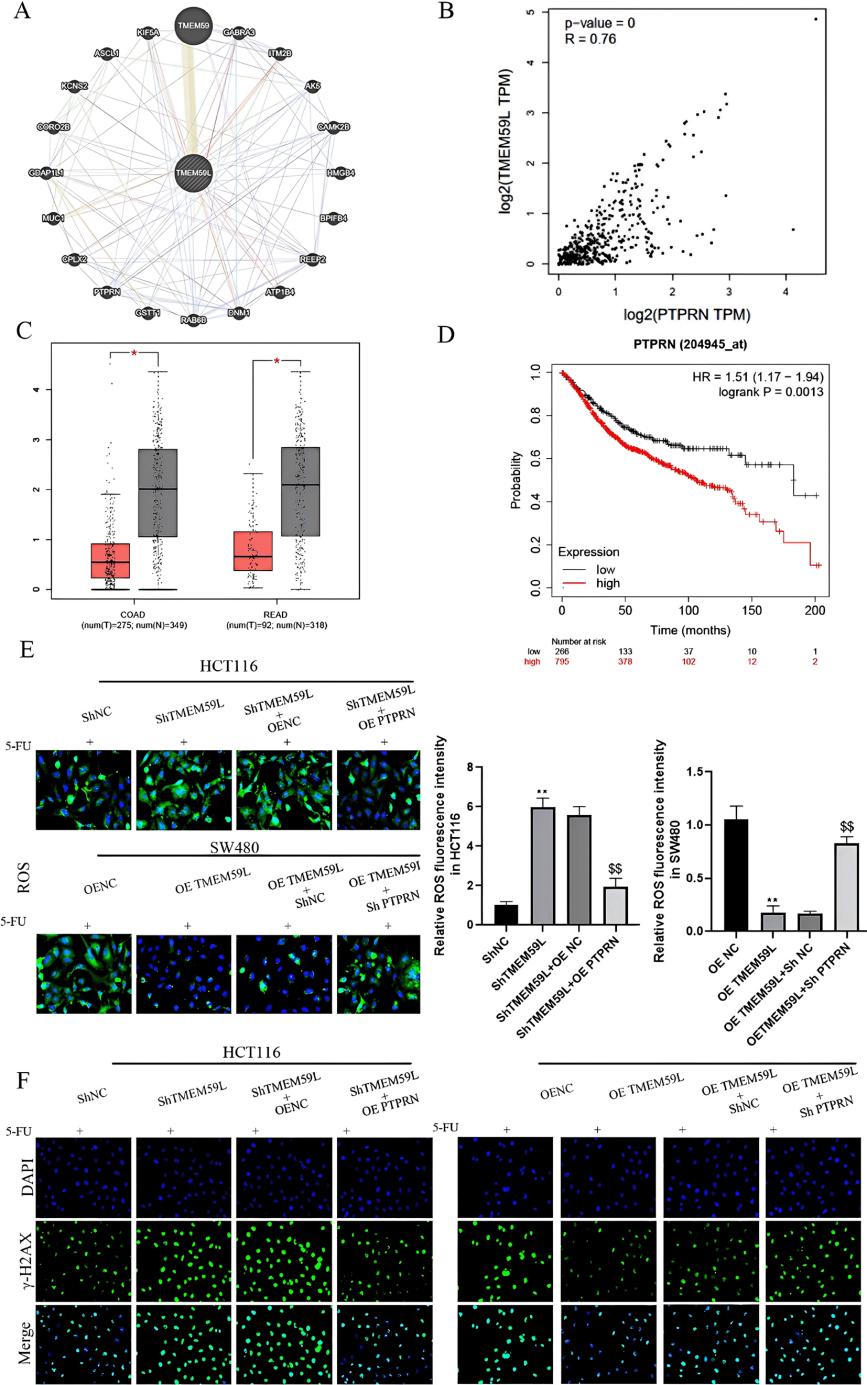
TMEM59L regulated 5‐FU induced DNA damage and ROS through PTPRN. (A) Physical interactions with TMEM59L in GeneMANIA website. (B) Correlation analysis between TMEM59L and PTPRN in COAD from GEPIA database. (C) PTPRN expression in COAD and READ from the TCGA database analyzed by the GEPIA database. (D) Higher PTPRN expression was related to poorer OS in CRC patients from TCGA through Kaplan–Meier Plotter database. (E) PTPRN partially reversed the effect of TMEM59L on 5‐FU induced ROS of HCT116 and SW480 cells. (F) PTPRN partially reversed the effect of TMEM59L on 5‐FU induced DNA damage of HCT116 and SW480 cells.

To explore the molecular mechanism underlying TMEM59L function, we performed bioinformatics analysis and identified a correlation between TMEM59L and PTPRN expression in CRC tissues (Figure 5A,B). Like TMEM59L, PTPRN was downregulated in tumor tissues compared to normal tissues (Figure 5C), yet high PTPRN expression was associated with poor survival in CRC patients (Figure 5D). Functional studies showed that TMEM59L knockdown enhanced 5‐FU–induced ROS, an effect reversed by PTPRN overexpression. Conversely, TMEM59L overexpression suppressed ROS accumulation, which was restored by PTPRN knockdown (Figure 5E). Similarly, γ‐H2AX–based DNA damage assays indicated that TMEM59L knockdown enhanced 5‐FU–induced DNA damage in HCT116 cells, and this was mitigated by PTPRN overexpression. TMEM59L overexpression reduced DNA damage in SW480 cells, but PTPRN knockdown abolished this protective effect (Figure 5F). Further experiments in PTPRN‐depleted cells showed that modulating TMEM59L expression no longer affected DNA damage or ROS levels (Figures S1 and S2), indicating that PTPRN is required for TMEM59L‐mediated regulation of 5‐FU resistance in CRC.

### 3.6 The Effect of TMEM59L and PTPRN on DNA Damage, Apoptosis, Stemness and EMT In Vivo

**FIGURE 6:**
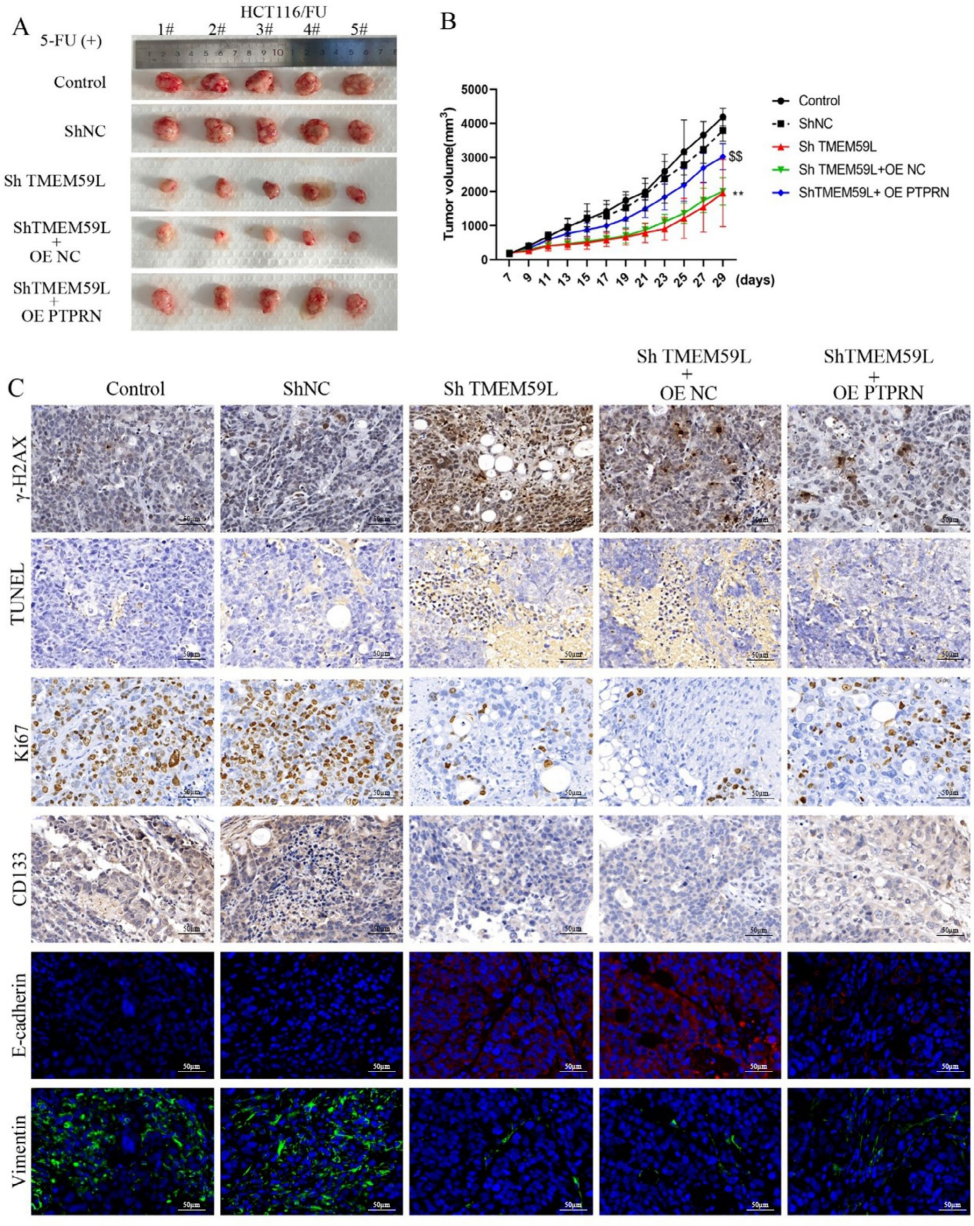
The effect of TMEM59L and PTPRN on DNA damage, apoptosis, stemness and EMT in vivo. (A) Images of Xenograft tumors from HCT116/FU cells. (B) Average tumor volumes are measured in xenograft mice every 2 days. (C) Representative images of γ‐H2AX, TUNEL, KI67, CD133, E‐cadherin and Vimentin in tumor tissues.

Silencing of TMEM59L expression enhanced the inhibitory effect of 5‐FU on tumor growth in mice with CRC, while overexpression of PTPRN reversed the impact of TMEM59L silencing on tumor growth (Figure 6A,B). Histological sections of mouse tumors indicated that downregulation of TMEM59L expression increased γ‐H2AX‐mediated DNA damage and E‐cadherin expression. TUNEL assay results showed that silencing TMEM59L expression enhanced apoptosis in colorectal cancer tumor cells (Figure 6C). The expression of Ki67, CD133, and Vimentin was significantly decreased in silence of TMEM59L group. The impact of TMEM59L downregulation on γ‐H2AX expression, apoptosis, Ki67, CD133, and EMT related markers such as E‐cadherin and Vimentin was attenuated by PTPRN overexpression in CRC bearing mice.

## 4 Discussion

The role of TMEM59L is different in several types of cancers such as GBM and COAD [5, 6]. In the present study, the action of TMEM59L in CRC was then explored. Interestingly, our data indicate that TMEM59L is expressed at lower levels in primary colorectal cancer tissues compared to adjacent normal tissues, but shows a marked increase in metastatic lesions. Paradoxically, high TMEM59L expression correlates with poor prognosis. This seemingly contradictory expression pattern may reflect the dynamic regulatory role of TMEM59L during tumor progression. This surprising finding is consistent with another study [5]. We speculate that TMEM59L expression is context‐dependent and stage‐specific: its initial downregulation in early tumorigenesis might reflect a loss of normal epithelial regulation, while its reactivation in metastatic stages may endow tumor cells with enhanced survival, chemoresistance, or stemness properties. Such biphasic expression patterns have been observed in other transmembrane proteins and may be shaped by factors like tumor microenvironmental stress, hypoxia, epigenetic reprogramming, or specific transcriptional regulators that shift during progression. Our results were similar to another study that TMEM59L may be an independent prognostic factor related to lymphatic metastasis of CRC [7]. The survival and proliferation of tumor stem cells are the main factors of drug resistance and relapse in tumor therapy [13]. Our results of the followed study showed that the downexpression of TMEM59L suppressed CRC cell proliferation, migration, and invasion along with EMT‐related E‐cadherin expression, while the overexpression of TMEM59L promoted cell proliferation, migration, invasion, and EMT‐related Vimentin expression. Given TMEM59L's localization to the plasma membrane and intracellular vesicular compartments, its role in chemoresistance may also involve modulation of membrane transport dynamics or regulation of signaling pathways linked to membrane‐associated drug efflux systems.

Chemotherapy resistance closely correlates with the prognosis of patients with CRC. It was found in our study that TMEM59L expression was upregulated in chemotherapy drugs such as 5‐FU, oxaliplatin, and capecitabine‐resistant patients with CRC and CRC cells. DNA damage and DNA damage repair play an important role in tumor therapy, including chemotherapy and radiotherapy. Drug resistance in cancer can arise from abnormalities in DNA repair pathways [14]. Exposure to platinum‐based chemotherapeutics or radiation often triggers the activation of these repair mechanisms, allowing cancer cells to survive and continue proliferating despite treatment, thereby reducing therapeutic effectiveness [15]. DNA damage response and repair genes associated with colon adenocarcinoma and rectal adenocarcinoma were obtained from recent bioinformatics analysis of TCGA data [16]. As ROS is involved in DNA damage [6], low expression of TMEM59L correlated with autophagy‐mediated attenuation of oxidative stress damage [17]. As one of the chemotherapy drugs, 5‐FU resistance may develop due to impaired drug uptake, target alterations, activation of DNA repair pathways, and resistance to apoptosis [18]. In the present study, it was found that TMEM59L was elevated and reduced 5‐FU sensitivity in 5‐FU‐resistant CRC cell lines while silencing TMEM59L expression increased ROS levels along with enhanced DNA damage and 5‐FU sensitivity in primary or drug‐resistant CRC cells. Previous studies have shown that 5‐FU‐resistant colorectal cancer cells exhibit elevated expression of stemness‐associated markers, including NOTCH1, CD44, ALDHA1, Oct4, SOX2, and Nanog. These resistant cells also demonstrate enhanced abilities in sphere and colony formation, as well as increased migratory and invasive potential [19] which was consistent with our study. Moreover, our results indicated that downregulation of TMEM59L downregulated the expression of CD133, Ki67, vimentin induced by 5‐FU resistance in CRC cells.

Bioinformatics analysis was performed to explore the molecular mechanism of TMEM59L in CRC, results of which showed PTPRN is a correlated marker with TMEM59L. As shown in a previous study, high expression of PTPRN inhibits NK cell cytotoxicity and promotes lung adenocarcinoma metastasis [20]. mRNA and protein expression of PTPRN was induced by hypoxia in the MCF10A, MDA‐MB‐231, and HCC1937 cell lines [21]. In our study, results demonstrated that overexpression of PTPRN attenuated the effect of TMEM59L silence on ROS combined with DNA damage, cell proliferation, apoptosis, metastasis, and cancer stem cells marker in CRC cells and mice bearing CRC tumor. Our results were consistent with a previous study that PTPRN was associated with invasiveness, metastasis, and EMT in CRC [11].

In summary, our study uncovers a dual role for TMEM59L in CRC: while downregulated in primary tumors, it is reactivated in metastases to drive chemoresistance—a phenomenon potentially regulated by tumor microenvironmental cues. By linking TMEM59L to PTPRN, we propose a novel axis wherein TMEM59L enhances DDR via PTPRN, enabling CRC cells to evade 5‐FU‐induced apoptosis. This mechanism contrasts with prior reports in glioblastoma, underscoring tissue‐specific roles for TMEM59L. Clinically, our findings highlight TMEM59L/PTPRN as actionable targets to counteract 5‐FU resistance. Future investigations should focus on identifying the upstream regulators of TMEM59L in CRC, particularly in metastatic versus primary lesions, and clarify how these regulatory networks converge on TMEM59L to modulate its function across different tumor stages—a limitation acknowledged in this work.

In conclusion, these findings suggest that TMEM59L, as a membrane transport‐associated protein, may serve as a novel target for modulating drug resistance in CRC through membrane signaling and DNA repair pathways.

## Author Contributions

W.J., Q.M., and Z.M. conceived and designed the project. W.J., Q.M., J.H., and F.W. carried out the experiments. Z.M., H.K., and C.W. participated in the data analysis. W.J. and Q.M. wrote the manuscript. F.L. and X.W. revised the manuscript. W.J. and F.L. confirm the authenticity of all the raw data. All authors read and approved the final manuscript.

## Funding

This work was supported by Inner Mongolia Science & Technology Plan.

## Ethics Statement

Ethical approval for the use of human tissue samples was granted by the Ethics Committee of Pudong Hospital Affiliated to Fudan University, with all participants providing written informed consent. All research procedures adhered to the principles outlined in the Declaration of Helsinki. Ethical approval number: QWJWLX‐01.

## Consent

Written informed consent has been obtained from the patients to publish this paper.

## Conflicts of Interest

The authors declare no conflicts of interest.

## Supporting information


**Figure S1:** The effect of TMEM59L knockdown or overexpression on ROS production in PTPRN knockdown cells.
**Figure S2:** The effect of TMEM59L knockdown or overexpression on DNA damage in PTPRN knockdown cells.

## Data Availability

The data that support the findings of this study are available from the corresponding author upon reasonable request.
