# Supplementary material for: Transmembrane Protein TMEM59L Modulates 5‐FU Resistance via PTPRN‐Mediated DNA Damage Repair in Colorectal Cancer
Source: Cancer Rep (Hoboken). 2026 Jan 16;9(1):e70448. doi: 10.1002/cnr2.70448 (PMC12809716; doi:10.1002/cnr2.70448)
Supplement: Supplementary file 1 — Figure S1: The effect of TMEM59L knockdown or overexpression on ROS production in PTPRN knockdown cells. Figure S2: The effect of TMEM59L knockdown or overexpression on DNA damage in PTPRN knockdown cells. [file CNR2-9-e70448-s001.docx]

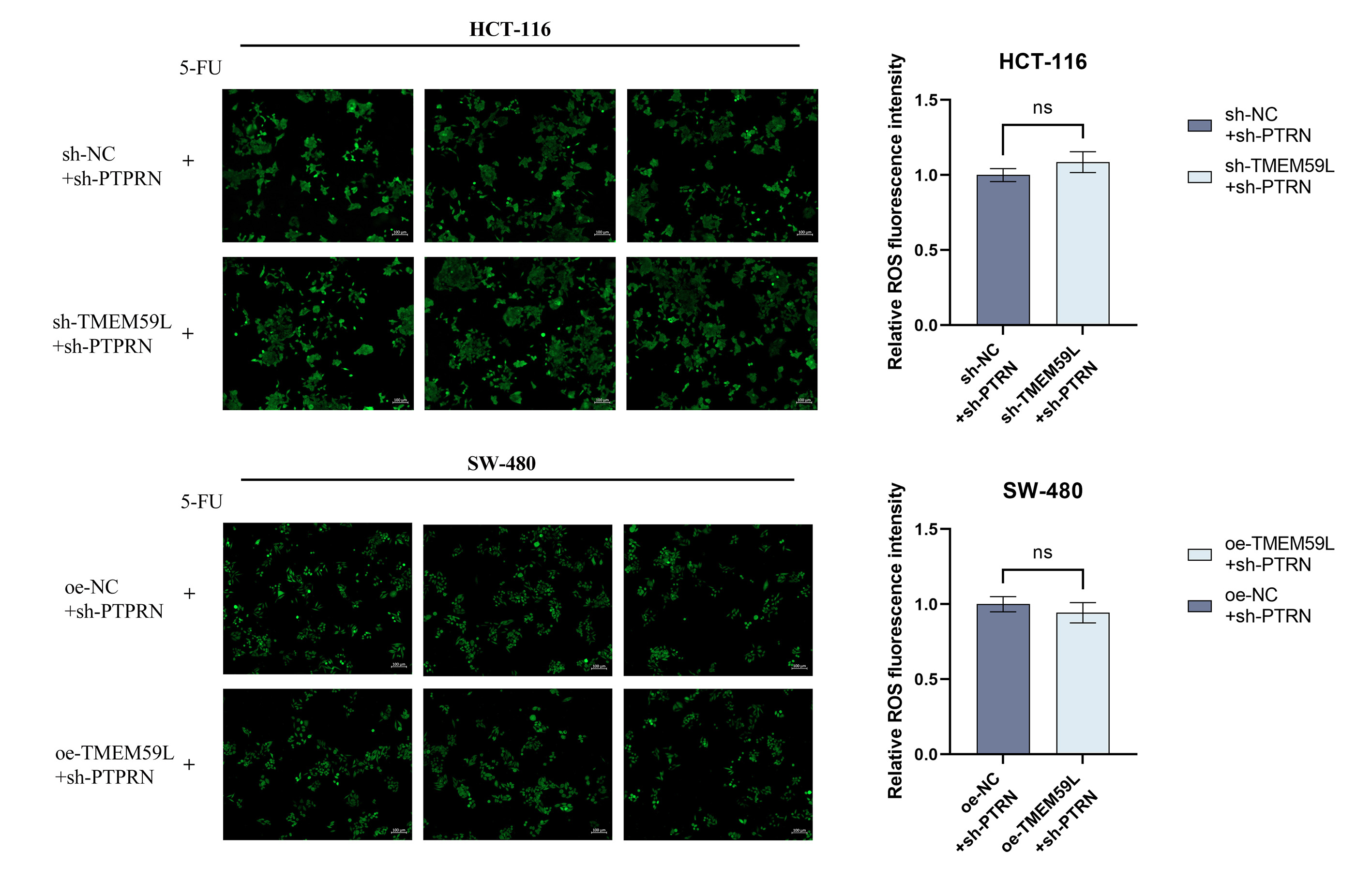


**Figure S1**. The effect of TMEM59L knockdown or overexpression on ROS production in PTPRN knockdown cells.


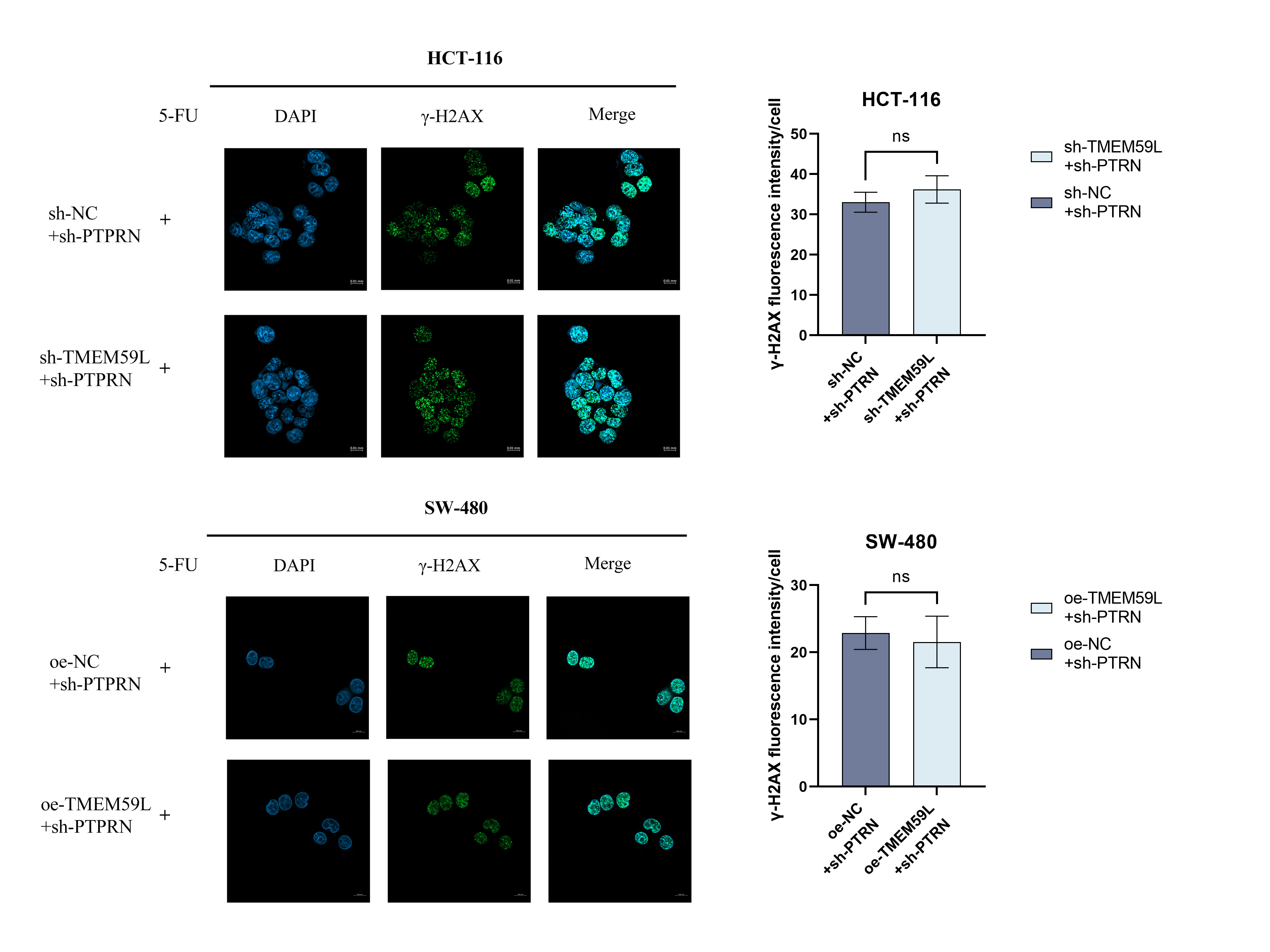


**Figure S2**. The effect of TMEM59L knockdown or overexpression on DNA damage in PTPRN knockdown cells.
